# Identification of Effective Programs to Improve Access to and Use of Trails among Youth from Under-Resourced Communities: A Review

**DOI:** 10.3390/ijerph17217707

**Published:** 2020-10-22

**Authors:** Julian A. Reed, Rachel M. Ballard, Michael Hill, David Berrigan

**Affiliations:** 1 Health Sciences, Furman University, Greenville, SC 29613, USA; 2 Prevention Research Coordination, Office of Disease Prevention, NIH, Bethesda, MD 20892, USA; rachel.ballard@nih.gov; 3 Landscape Architect, Enterprise Program, U.S. Forest Service, Washington, DC 20250, USA; michael.hill1@usda.gov; 4 Behavioral Research Program, Division of Cancer Control and Population Sciences National Cancer Institute, 9609 Medical Center Drive MSC 7344, Bethesda, MD 20892-7344, USA; berrigad@mail.nih.gov

**Keywords:** recreational trails, trail interventions, children and youth physical activity on trails

## Abstract

The primary purpose of this paper is to identify and review studies evaluating the effectiveness of programs to increase access to trails and trails use (physical activity) among youth from under-resourced communities. Three additional goals include identifying: (1) Correlates of physical activity/trail use and features of transportation systems and/or built environment and land use destinations, that may inform and support the planning and implementation of programs to promote trail use among youth, (2) benefits associated with trail use, and (3) barriers to trail use. Under-resourced communities are defined as those lacking sufficient resources (i.e., under-funded). METHODS: A review of the literature was conducted to identify, abstract, and evaluate studies related to programs to promote trail use among youth and youth from under-resourced communities. In anticipation of very few studies being published about this topic, studies were also reviewed to identify correlates of transportation systems and built environment and land use destinations related to increases in physical activity, and benefits of, and barriers to trail use. PUBMED, MEDLINE, PsycINFO, Sportdiscus, Annual Reviews, American Trails, and Google Scholar databases were searched using terms including trails, built environment, physical activity, exercise, walking, children, adolescents, and youth to identify studies that potentially related to the purposes for conducting this review. Review methods identified, 5278 studies based on our search terms. A review of study titles, abstracts, and select full article screens determined that 5049 studies did not meet the study inclusion criteria, leaving 221 studies included in this review. RESULTS: No studies were located that evaluated programs designed to promote and increase trail use among youth, including youth from under-resourced communities. Eight studies used longitudinal or quasi-experimental designs to evaluate physical activity and neighborhood characteristics prospectively among adolescent girls (*n* = 1), the effects of the path or trail development on physical activity behaviors of children, youth, and adults (*n* = 4), marketing or media campaigns (*n* = 2), and wayfinding and incremental distance signage (*n* = 1) to promote increased trail use. Correlates of transportation systems (e.g., trail access, road traffic congestion related to safe active travel, lack of sidewalks, closer proximity to trails, access to transportation), destinations (e.g., park availability and access, park improvements, greenspaces), or both routes and destinations (e.g., perceptions of safety, lighting), were identified. These correlates may support the planning and implementation of programs to increase trail use among youth, or may facilitate the connection of trails or routes to destinations in communities. Barriers to trail use included costs, crime, lack of transportation, lack of role models using trails, and institutional discrimination. Conclusions: Scientific evidence in support of addressing the underrepresentation of trail use by youth from under-resourced communities is lacking. However, there is a related body of evidence that may inform how to develop programs that support trail use by youth from under-resourced areas. Dedicated, deliberate, and systematic efforts will be required to address research and knowledge gaps, and to evaluate programs and practice related to trail use among youth from low income, often racially or ethnically diverse under-resourced neighborhoods or communities.

## 1. Introduction

Outdoor trail use is a health-enhancing behavior with the significant potential to increase physical activity, active commuting and exposure to green environments in youth. Levels of trail use by youth are low overall and even lower by youth from under-resourced, low income, and communities of color [1]. Youth from such communities could benefit greatly from the physical, mental/emotional, and social benefits of trail use. Although we are most interested in trail use as a mode of recreational physical activity, for the purposes of this review we included studies that evaluated outcomes related to both increases in physical activity, and/or trail use as a type of physical activity specifically. We include papers with physical activity as an outcome in addition to trail use per se because of the pressing need to identify approaches to increasing physical activity in children and adolescents.

A considerable body of evidence suggests that improvements to the built and natural environments via enhanced walkability, parks and trail infrastructure can increase physical activity, fitness and health across a broad range of the population from children to adults. Studies of adults show that the benefits of active commuting, walking and cycling, on health, include lower rates of cardiovascular disease and cancer compared to adults who are not active commuters [2]. Spending two hours in nature (parks, woodlands, or on beaches) has also been found to be significantly associated with improvements in mental health, such as improved psychological well-being [3]. Researchers have also found that a 20-min session in nature is linked to stress reduction as measured by salivary biomarkers [3]. The evidence available suggests active travel interventions can increase walking among children, limited evidence exists, however, linking active travel to school to overall daily activity. [4]. However, little is known about the physical and psychological benefits associated with active commuting or walking and cycling for recreation regarding children and youth. Additionally lacking is understanding how to engage youth from under-resourced, often low-income and diverse communities to use trails for recreation, to potentially promote their physical, mental, and social health and well-being.

The primary aim and objective of this review paper is to identify trail studies that may effectively promote and increase the use of trails among youth, especially those from under-resourced neighborhoods or communities. Three additional goals of the review include identifying: (1) Correlates of physical activity/trail use and features of transportation systems and/or built environment and land use destinations, that may inform and support the planning and implementation of programs to promote trail use among youth, (2) benefits associated with trail use, and (3) barriers to trail use.

### 1.1. Physical Activity/Inactivity, Fitness and Health

In the United States (U.S.), and many countries worldwide, levels of physical activity are lower than recommended levels and the amount of sedentary time is increasing in children and youth [5,6,7,8]. Lack of adherence to such recommendations is associated with lower fitness levels, increased BMI, reduced strength and unhealthy body composition [5,6,7,8]. In the U.S. and other developed countries, under-resourced, low-income and minority communities suffer from disparities in fitness and weight status [6,9].

Overall, physical inactivity has been associated with lower levels of aerobic fitness and “is a cause of chronic disease in children and adolescents” [5]. In the U.S., only 42% of adolescents ages 12–15 are meeting standard levels of cardiorespiratory fitness, a 10% decrease from a decade ago [6]. Recent findings indicate lower levels of aerobic fitness are associated with higher rates of overweight and obesity [1,2,3,4,5,6,7,8,9,10,11,12,13]. The 2018 Guidelines Advisory Committee Scientific Report in the U.S. found that for prospective research, physical activity was inversely associated with overweight/obesity [12]. Currently, 25 million American children are overweight or have obesity considered overweight or obese, and are more likely to be obese in adulthood if physical activity behaviors do not improve in adolescence [14,15]. Lack of sufficient muscular strength, muscular endurance, and aerobic capacity reduced the ability of children to achieve physical activity associated health benefits [7,8,9,10,11]. Public health problems associated with less fit youth and overweight or obese are also characterized by disparities among different population demographic groups. African American and Hispanic children and adolescents have substantially higher levels of overweight and obesity compared to their Caucasian counterparts, placing racial/ethnic minority populations at a greater risk of health complications later in life [15,16,17,18]. Therefore, it is important for children and youth to have access to supportive environments and programs that facilitate the use of trails to promote outdoor recreational opportunities, exploration of nature, and increases in physical activity [19,20,21,22,23]. However, efforts to increase physical activity among youth to address fitness and weight disparities will need to be inclusive of, and tailored for, youth from under-resourced neighborhoods or communities. Programs tailored to the needs and preferences of youth from under-resourced areas, should address barriers and facilitate the use of trails among youth living in these areas.

### 1.2. Trails, Built Environment Features, Physical Activity and Health

Community infrastructure is often considered a foundation for health and wellness that may affect planning and land-use decisions that may also be related to greater physical activity and improved health outcomes [24,25,26]. Researchers have identified trails, as part of the integral infrastructure for physical activity [27,28,29], and trails have been found to be associated with regular physical activity participation [30,31]. Researchers have also recommended pedestrian or bicycle routes connect with destinations to promote physical activity for transportation [23,30,31,32,33,34] and leisure physical activity. Access to recreational trails is widely accepted to influence physical activity participation among varying populations including children and youth [35,36,37,38,39,40,41,42,43,44,45].

Several approaches are being taken to understand the importance of trails to support physical activity and enhance health. Reed and colleagues examined activity behaviors in 25 parks and found trails to be the most frequently used feature in the parks [46]. Sixty-percent of adult males and 81% of adult females observed in all 25 parks were on trails. The South Carolina Trails Survey highlighted by American Trails, however, found few children and youth using a prominent rail-trail conversion [47]. Trail Development and increased access to trails may promote regular physical activity. Librett and colleagues [48] examined the physical activity levels among trail users in the U.S., and found that individuals who reported using trails at least once a week were twice as likely to meet physical activity recommendations as individuals who reported rarely or never using trails. Hughey and colleagues [24] examined the associations between adults’ use of a paved trail, their weight status and self-rated health and found that trail users were half as likely to be overweight or obese as trail nonusers. Trail users were also significantly more likely to report high self-rated health than nonusers. Kaczynski and colleagues [49] found that park features (e.g., trails and/or paths in a park, playgrounds), contributed to participation in physical activity, and trails had the strongest relationship with activity participation of all park features in their study. A review article by Mitten et al. identified numerous health benefits associated with hiking behavior. Benefits included increased time spent in social contacts, and enhanced mental, emotional, and physical health. These benefits may be acute such as reduced blood pressure and stress, or improved immune system functioning and restored attention. Chronic benefits derived from hikers over time have included weight loss, reduced depression, and greater wellness [25]. This body of work is consistent with the idea that trails could increase physical activity levels, however, most of these studies are cross-sectional.

Nevertheless, the U.S. Community Preventive Services Task Force (Task Force) released a recommendation on the influence of the built environment on physical activity [50]. The Task Force recommends, based on 90 studies (16 longitudinal and 74 cross-sectional), “built environment strategies that combine one or more interventions to improve pedestrian or bicycle transportation systems with one or more land use and environmental design interventions to increase physical activity.” The Task Force further recommended that “Coordinated approaches must combine new or enhanced elements of transportation systems with new or enhanced land use and environmental design features. Intervention approaches must be designed to enhance opportunities for active transportation, leisure-time physical activity, or both.” The Task Force recommendations pertain to all ages and include children and adolescents. However, there is scarce evidence about how to promote and optimize the use of routes connected with destinations especially among youth and disparate populations such as different racial/ethnic groups. Considerations about “how to” tailor interventions to promote and increase trail use among disparate groups are especially important, since education and income are both positively associated with trail use [41]. Greater efforts are needed to specify how connectivity can best be addressed in support of increased trail use for under-resourced communities.

### 1.3. Trails or Other Built Environment Features, and Physical Activity among Youth from Under-Resourced Communities

Multi-use recreational trails influence adult physical activity participation [23,30,35,36,37,48,51,52]. An understudied research topic is the impact of trails and other built environment interventions on the physical activity behaviors of children and adolescents including those living in under-resourced (also referred to as “underserved” in the literature) neighborhoods or communities [53,54,55,56,57,58,59,60,61]. The use of the terms under-resourced children and youth in this paper refers to those who are living in under-resourced communities. These communities are often predominantly made up of residents from diverse racial, ethnic, and cultural backgrounds, who also typically lack the access to health care, economic, financial, and social benefits accruing to people from more affluent backgrounds and communities.

Community and built environments to promote physical activity earned a “C” from the 2018 U.S. Report Card on Physical Activity for Children and Youth (“The Report Card is a resource that summarizes health statistics related to physical activity levels among children and youth in the U.S. More importantly, the Report Card is an advocacy tool that provides a level of accountability and call-to-action for decision-makers regarding how we, as parents, teachers, health professionals, community leaders, and policymakers, can implement new initiatives, programs, and policies in support of healthy environments to improve the physical activity levels and health of our children and youth.”). This grade decrease from a B—in the two previous evaluations was primarily related to children and youth having barriers to access to parks and other recreation facilities. The 2018 Report Card considered new elements of the community and built environment such as: safety, walkability of the community, and complete streets policies [62]. These additional elements were the key reason for the lowered grade. Nonetheless, the Report Card highlighted the importance of improving the community and built environments to support child and youth physical activity, with a focus on underserved (under-resourced) communities [62]. Considering less than one-third of U.S. States report having at least 30% of youth residing in high walkable communities, improving these environments is essential [63] including creating or improving trails and trail connectivity.

Survey data indicate that members of under-resourced communities are underrepresented among trail users [64]. The National Visitor Use Monitoring (NVUM) survey [64], an ongoing questionnaire administered to U.S. Forest Service park users over a 10-year timeframe, indicates that 95% of people engaged in recreation on U.S. Forest Service lands are White; 6% are Spanish, Hispanic, or Latino; about 3% Asian; 2% American Indian/Native Alaskan; and only 1% are Black or African American (respondents could choose more than one racial group). This contrasts with the overall race/ethnic composition of the U.S. which was 61% Non-Hispanic White, 15.3 Hispanic, 13.4% Black or African American, 5.9% Asian with the remainder Native American, Alaska Native, Native Hawaiian or two or more races as of 2019. Survey findings also indicate that youth, age 16 to 19 years, recreate on U.S. Forest Service lands less than any other age group (about 4%), except for adults 70 years and older (5%). Although U.S. Forest Service trails survey data may not be representative of trail users in more urban or even rural parts of America, the existing evidence indicates that youth, especially youth from diverse backgrounds and cultures, are underrepresented among trail users [22].

To address this gap in knowledge, the National Collaborative on Childhood Obesity Research (NCCOR)—a public-private partnership among the National Institutes of Health, the Centers for Disease Control and Prevention (CDC), the Robert Wood Johnson Foundation, and the U.S. Department of Agriculture, along with collaboration for this project from staff from the Federal Highway Administration formed a scientific workgroup of member organizations and partners to investigate effective interventions and programs for increasing trail use among children and youth from under-resourced communities. NCCOR also engaged a Principal Investigator (PI) (author Julian A. Reed) to conduct the review, with feedback from the workgroup. Methods used to conduct the review, results, and discussion of findings are highlighted next.

## 2. Methods

A review of the peer-reviewed literature was conducted to identify efficacious and effective programs for increasing trail use (urban, nonurban, and/or more remote trail use) among children and youth of all abilities who are from underserved/under-resourced communities. Studies were also reviewed to identify correlates of physical activity/trail use and features of transportation systems and/or built environment and land use destinations. For this review, the PI searched health, medical, public health and sport sciences journal databases in PUBMED, MEDLINE, PsycINFO, Sportdiscus, Annual Reviews, American Trails, and Google Scholar from 2000–2018 for research related to trail use and physical activity by children and youth with a primary focus on intervention studies. These databases identified publications in health, medical, public health and sport sciences fields with a focus on peer-reviewed, primarily, journal articles including abstracts and studies related to our topics of interest.

**Figure 1:**
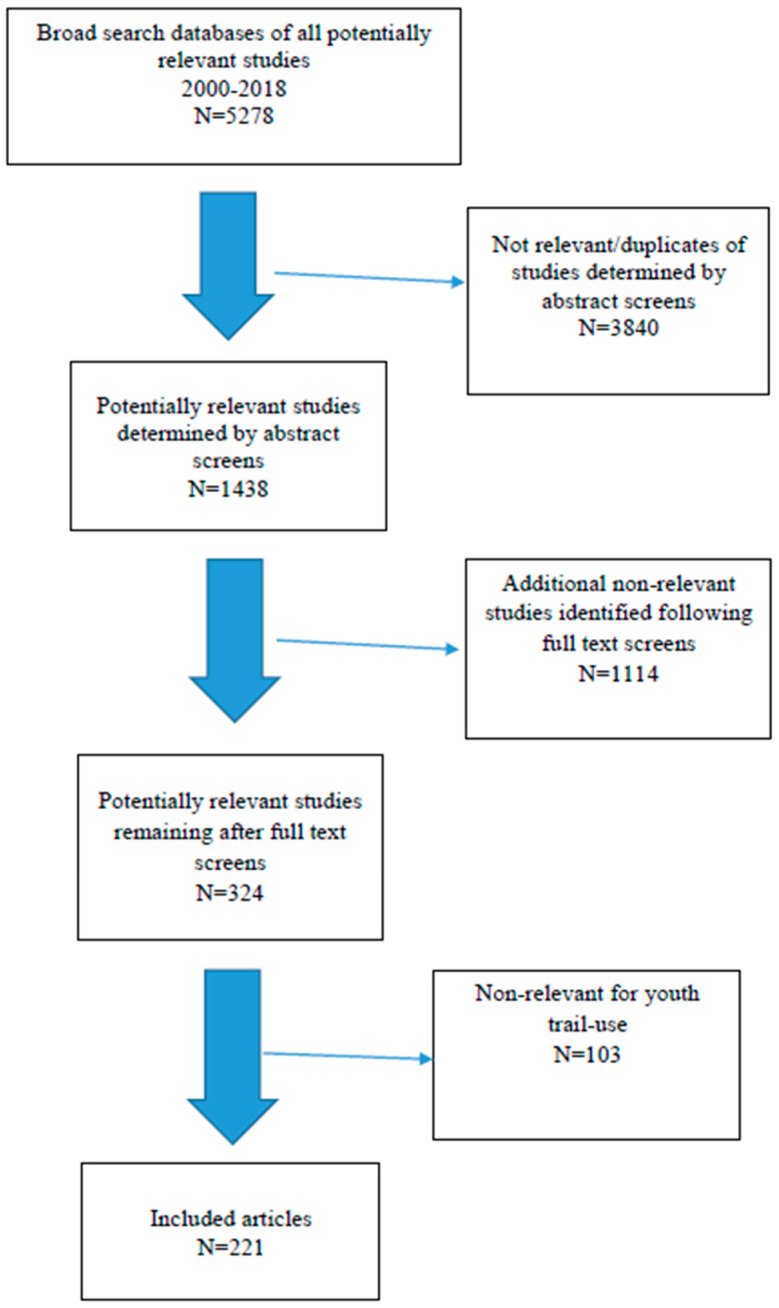
Flowchart of inclusions/exclusions for articles selected for review.

Eligibility criteria included papers addressing: (a) children and youth (ages six to 17) and physical activity in the built environment(s) with a primary focus on pedestrian or multi-use trails (i.e., trails, trails in parks, rails-to-trails, sidewalks, trails in greenspaces, walking paths, neighborhood trails, city trails), and (b) underserved children, and adolescents and built environment, trails and physical activity. Although we prefer to use the term under-resourced rather than underserved regarding our priority population, we used underserved as a search term, because it is a term that has been used frequently in other studies). The references of all reviewed articles were also examined to identify other studies related to our review (see Figure 1). More than 50% of all abstracts identified were cross-referenced by the primary investigator and at least one other member of the research team. All (*n* = 324) selected papers for potential relevance were reviewed by two or more research team members and the primary investigator.

A combination of the following search terms was used: “Trails”; “Trails and Physical Activity”; “Trails and Children and Youth”; “Built Environment and Adolescents, Youth”; “Children and Youth, Adolescent Physical Activity and Built Environment”; “Exercise and Built Environment”; “Physical Activity and Open Spaces”; “Open Spaces and Adolescents and Youth”; “Recreational Facilities and Youth Physical Activity”; “Walking Trails”; “Walking Trails and Youth and Adolescents”; “Active Transportation and Youth”; “Child Physical Activity”; “Adolescent Physical Activity”; “Urban Trails and Physical Activity”; “Rural Trails and Physical Activity”; “Rural Trails and Child Physical Activity”; “Greenspace and Physical Activity”; “Built Environment Design and Physical Activity”; “Public Facilities and Physical Activity”; “Intervention Studies and Physical Activity, Children, Youth, Adolescents”.

## 3. Results

The search strategy initially identified 5278 potential articles from the literature search (Figure 1). Of these, 221 eligible studies were selected for full abstraction and inclusion in this review. The vast majority of studies located were correlational in nature. However, eight studies used prospective, longitudinal, or quasi-experimental designs [32,65,66,67,68,69,70,71] evaluating increased availability of trails [65,71] or other neighborhood characteristics [67] with physical activity/trail use, and media campaigns [32,66] or informational strategies to promote and increase trail use [66]. Of these eight studies, one focused solely on adolescent girls [67], and is reviewed in more detail below. Two additional studies [68,69] are also highlighted below as examples of the types of longitudinal research needed to advance the science and knowledge related to youth trail use. The remaining five studies [32,65,66,71] focusing on adults or adults and youth combined are also excellent examples of prospective studies related to trails and trail use.

**Figure 2:**
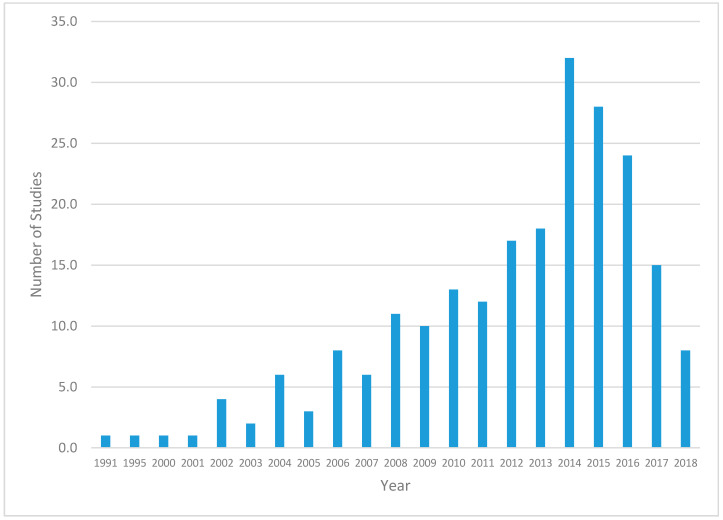
Publication date of the papers considered in this review.

**Figure 3:**
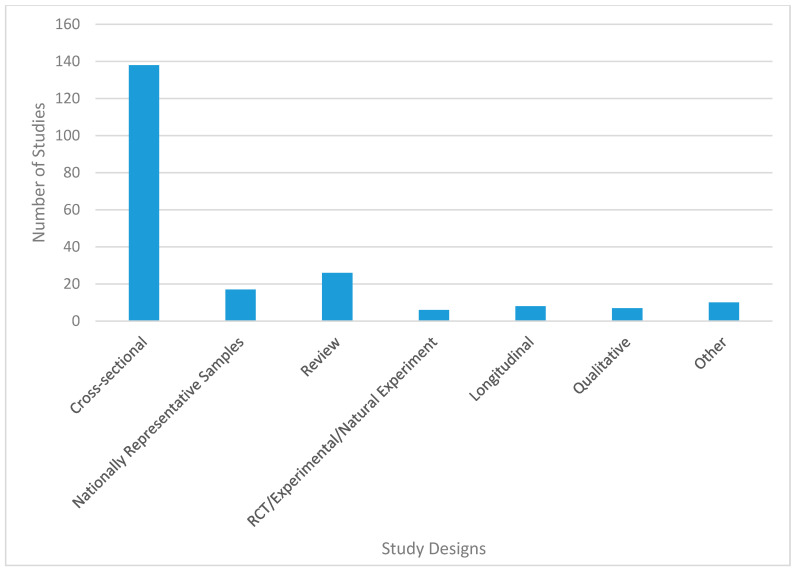
Distribution of studies considered in this review by study design.

**Figure 4:**
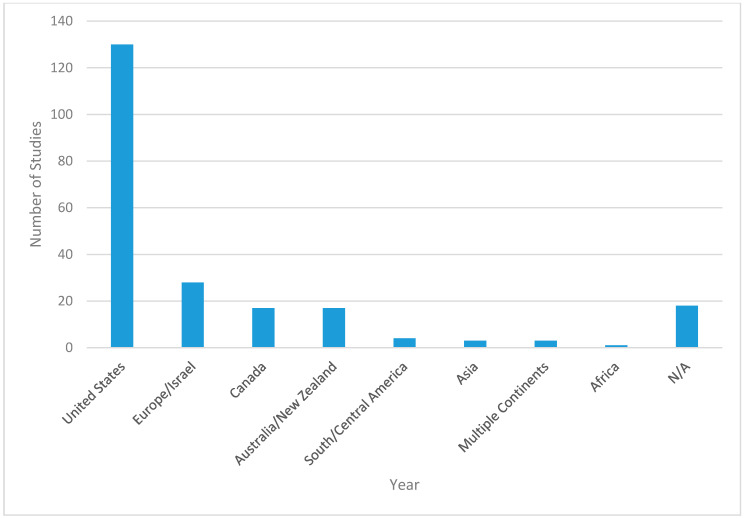
Countries/Continents on which the studies considered in this review were carried out. Reviews were categorized as not applicable (N/A).

**Figure 5:**
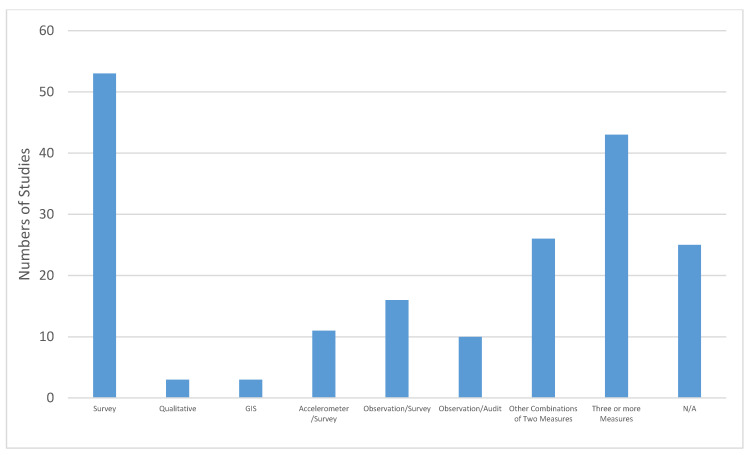
Measurement approaches used in the studies considered in this review were carried out. Reviews, policy statements and study design paper were categorized as not applicable (N/A).

Figure 2, Figure 3, Figure 4 and Figure 5 provide further details of the papers selected for full abstraction. Of the 221 papers considered in this review, published from 1991 to 2018 (Figure 2) the majority (Figure 3) were cross-sectional analyses of convenience or locally representative samples (*n* = 138). The remaining studies included analyses of nationally representative samples [17], reviews [26], quasi-experimental or longitudinal [8] and a small number of experimental studies, policy statements, study designs and methods research. Most studies occurred in the United States (Figure 4). Figure 5 illustrates the measurement approaches used in the studies underlying the review. More than half of the studies use multiple measurement approaches, and more than 40 studies use three or more measurement approaches, highlighting the need for multi-disciplinary teams to investigate trail use and its determinants.

### 3.1. Study Primary Purpose: Effective Programs for Increasing Trail Use among Children and Youth from Under-Resourced Communities

The results indicate that there are no published experimental studies that have evaluated the effectiveness of programs designed to encourage or increase the use of trails by youth from under-resourced communities. However, some longitudinal evidence exists on the influence of neighborhood characteristics and transport with 2-year changes in accelerometer-assessed non-school moderate- to vigorous-intensity physical activity, and sedentary behavior in a sample of adolescent girls from diverse race/ethnic groups (53.5% White; 18% Black; 19.1%; Hispanic; 4.8% Asian, Native Hawaiian or Pacific Islander [67].

Physical activity behavior typically declines with adolescents with age [67,72]. For this study, the researchers hypothesized “that girls who perceived a more conducive neighborhood environment for physical activity, more physical activity opportunities, and better transport options at baseline would have more favorable changes from 6th to 8th grade in non-school physical activity and sedentary behavior”. This study, however, did not find that neighborhood features such as good lighting and trails were prospectively related to increases in physical activity at 2-years follow-up. Rather neighborhood features were associated with decreases in non-school Met-weighted Moderate- to Vigorous-intensity Physical Activity (MW-MVPA) at follow-up. No neighborhood measures were associated with sedentary behavior [67]. The researchers indicate that there may be two possible reasons for their findings. First, it may be that girls’ who reported common physical activities in 6th grade shifted to doing more sedentary behaviors such as talking on the phone and music lessons in middle school that replaced popular physical activities done during elementary school. Second, perhaps relationships among the environment and physical activity change as children age for reasons other than doing preferred types of physical activities. Third, children who engage in physical activity outdoors may be more likely to notice dangerous locations and unpleasant smells in their neighborhood, that actually impede activity, because outdoor activity, gives them greater exposure to these characteristics than peers who are less active.

In addition to the prospective study above, cross-sectional and observational studies have evaluated associations between environmental infrastructure and neighborhood characteristics and physical activity among youth from diverse backgrounds or groups [73,74,75,76,77,78]. One study [21] surveyed diverse youth (White, 48.5%; Black, 18.7%; Hispanic, 14.1%, Asian/Pacific Islander, 3.1%) to evaluate the associations between their perceptions of environmental factors and transportation with their physical activity and active transport to school behaviors. Perceptions of neighborhood walking trails, ease of walking or biking to transit, destinations within walking distance from home, neighborhood safety, aesthetics (more trees, interesting things to look at, lack of garbage or litter), number of activity facilities, and parental support to walk or bike for transportation were all related to physical activity, and in some cases to active transport [21].

In another study evaluating the relationship between community design features and access to recreational facilities among a diverse population of adolescents (43% ethnic minority) [34], there was some evidence that community design and access to recreational facilities are associated with physical activity. Nearby recreation facilities and the number of nearby parks correlated positively, and intersection density, inversely, with girls’ physical activity. The amount of retail floor area correlated positively with boys’ physical activity. No design or recreational access features correlated with BMI percentiles. In another study, researchers examined the relationship between parental nativity with active transport to school, active use of sidewalks, use of local neighborhood parks, and use of neighborhood physical activity facilities, in an under-resourced neighborhood in New Jersey, and found Latino youth with foreign-born parents were generally more physically active than were Latino youth with parents born in the U.S. [73]. however out of the four areas evaluated (transport to school, use of parks, sidewalks, and facilities) findings were significant only for the active transport to school. Latino youth with parents living in the U.S. for less than 10 years were 1.5-times more likely to walk to school than the youth of U.S.-born parents. These findings suggest that parental nativity status may be an important influence on policies or interventions designed to increase physical activity among Latino youth. The authors state that “identifying the factors that seem to be health-enhancing among foreign-born populations,” may help inform and tailor interventions that can be effectively used with U.S.-born youth with foreign-born parents. In another study [74], a national Safe Routes to School survey was adapted to evaluate if, and how children and adolescent school students in a traditionally under-resourced, predominantly Latino community in East Los Angeles used an urban greenway for transportation to school. The findings suggest that the greenway pathway near an elementary school and high school made “it easier and safer for students to walk or bike to school compared, to using the often inadequate sidewalk and street infrastructures in this urban environment” [74]. Findings may inform the use of greenways for transportation in other communities. Another study focused on the disparities of under-resourced adolescents and physical activity resources in neighborhoods [75]. Data from the 2011–2012 National Survey of Children’s Health were analyzed to identify the demographic characteristics of youth, aged 10–17 years, who live in neighborhoods that are supportive of physical activity. Findings indicate that the proportion of youth living with access to built environment supports for physical activity was lower among non-Hispanic Black or Hispanic youth, those overweight or obese, from homes with lower socioeconomic status or from rural areas [75].

Two additional studies conducted outside the U.S. provide insights into the built environment and physical activity among youth from diverse or under-resourced areas. Cross-sectional relationships were examined between neighborhood-built environment and walkability characteristics, and socio-economic status, with multiple physical activity behaviors, sedentary time, and obesity indicators among adolescents from Valencia, Spain [76]. Moderate-vigorous physical activity was highest, and sedentary minutes lowest, in high-SES/high-walkable neighborhoods. Neighborhood SES was also positively related to participation in sports teams, and physical activity classes and, negatively related to time spent in sedentary behaviors. Adolescents living in lower-SES neighborhoods watched more TV and were more obese. Findings from this study indicate it is important to take into consideration the interaction of neighborhood built and SES environments when planning health promotion interventions for adolescents. In another study [77], the prevalence and correlates of active commuting to school (ACS) were examined in a nationally representative sample of Mexican adolescents, using data of adolescents ages 10–14 years, from the 2012 Mexican National Health and Nutrition Survey [77]. ACS was negatively associated with travel time, age, mother’s education level, household motor vehicle ownership, family socioeconomic status, living in urban areas, the North region of the country, and overweight/obesity. Each additional minute of ACS was associated with a 1% decrease in the odds of being overweight or obese.

The above studies related to neighborhood characteristics, the built environment and physical activity among youth from diverse and under-resourced communities are difficult to interpret, because of different methods and outcomes evaluated in the studies. However, one consistent finding is that SES is consistently positively related to physical activity, and inversely related to inactivity/sedentary behavior among youth, in the U.S. [75] and across cultures and other countries [78,79].

### 3.2. Study Secondary Purpose 1: Transportation Systems and Built Environment and Land-Use Correlates and Features that May Support Programs Designed to Provide Increased Opportunities for Physical Activity (i.e., Trail Use) among Youth

Cross-sectional studies and several reviews report that greater access to routes and features of destinations are associated with improvements in daily moderate to vigorous physical activity participation [26,29,57,61,76,79,80,81,82]. To date, these studies have not included a focus on diverse racial or ethnic groups or under-resourced or low SES neighborhoods or communities. Mitchell and colleagues [61], for example, found that boys and girls from neighborhoods with more access to parks having sports fields had significantly higher moderate- and vigorous-intensity physical activity. When the researchers accounted for individual and neighborhood socio-demographic variables, multi-use path space also favorably predicted moderate- to vigorous-intensity physical activity [61]. Another example, typical of this body of research, is an observational study that relied on direct observation of behaviors in 45 parks in a southeastern community. This study identified large numbers of children participating in physical activity on trails. Trails were located in 16 of the 45 parks (36%), and close to 10% of all boys and girls were observed using trails in those 16 parks [22]. Findings from these studies provide examples of trails supporting youth physical activity, and indicate that efforts to prioritize planning trail connectivity to recreational spaces, and facilities to support youth physical activity are important [83].

Many other cross-sectional studies have identified correlates of trails or other routes (e.g., paths, sidewalks, bicycle routes) and physical activity/trails use (see Table A1, Section A) for select examples). Overall, these studies illustrate that access to trails, paths, trails in parks, access to transportation, closer proximity to trails, road traffic-related to safe active travel, intersection density, and lack of sidewalks) are related to physical activity. These correlates may be important to consider in future interventions connecting trails to programs that promote trail use, and that are tailored for use with youth from diverse or under-resourced neighborhoods or communities.

In addition, many studies have identified correlates of destinations (e.g., parks, open spaces, playgrounds), and physical activity/trails use (see Table A1, Section B) for select examples). Overall, these studies illustrate that access to parks, greenspaces, open-spaces, and recreational facilities, and park enhancements (e.g., playgrounds), are also related to physical activity. These correlates may be important to consider during efforts to plan and develop effective programs to promote and increase trail use, outdoor recreational physical activity, and enjoyment of nature that are tailored for use with youth from diverse or under-resourced neighborhoods or communities.

Studies identifying correlates of both transportation systems and destinations and physical activity/trail use are also listed in Table A1 (Section C). These include favorable perceptions of safety, neighborhood size, neighborhood features (e.g., lighting, signage (e.g., distance from route to destinations), and mapping (wayfinding), as examples.

### 3.3. Study Secondary Purpose 2: Benefits Related to Trails Use

With the exception of one study [56], results did not lead to the identification of physical, mental/emotional, or social benefits of trail use by youth or adult trail users. McCracken, et al. [56] found that more access to greenspace was associated with better health-related quality of life, and self-esteem among children. Although the use of trails has been found to increase physical activity behaviors, our knowledge about other benefits of trails use is very limited, as highlighted in the subsection above titled, Trails or Other Built Environment Features, Physical Activity and Health.

### 3.4. Study Secondary Purpose 3: Barriers to Trail Use among Youth from Under-Resourced Communities

Results indicate that many youths from under-resourced communities lack exposure, experiences, and opportunities to access trails, and obtain benefits of trails use, due to a variety of social determinants of health (e.g., costs, lack of transportation, lack of role models using trails, and institutional discrimination) [50,83,84], crime [85,86] and automobile traffic [78,79,87]. Additional barriers to youth physical activity included schoolwork, weather conditions [88], and strangers [89].

The findings indicating that lack of seeing role models on trails is a barrier to trail use for some population groups, may be interrelated with findings from other studies. These include observations of park users in 45 parks that found a greater frequency and percentage of white youth compared to minorities [22], and a “major inhibitor” to park use that has been reported to include racial heterogeneity of the neighborhood [90]. Among Caucasian youth in Canada aged 8–10, perceptions of a high proportion of minorities in the surrounding area decreased feelings of safety related to physical activity. While for parents, high traffic and lack of perceived community involvement contributed to decreased feelings of safety [85].

Although much research has been published examining physical activity behaviors related to routes (e.g., trails, pathways), and destinations (e.g., parks, greenspaces), the majority of the studies have relied on correlational and cross-sectional designs that do not identify what works or how to intervene to be more inclusive of population groups who are underrepresented trail users. More robust study designs are needed to gain knowledge about programs that promote and increase trail use among youth from under-resourced communities, as are discussed next.

Few studies exist that specifically focus on the influence of trails and trail features on children’s and adolescents’ physical activity behaviors, and a lack of experimental research limits our understanding of how trails (routes) may be combined with program-based efforts (destinations) to increase trail use. Because changes in the built environment are rarely amenable to randomized controlled trials, observational studies of built environments, policies and programs that are not under investigator control, often termed “natural experiments”, may be needed to evaluate such changes. Evaluation of natural experiments with carefully selected control groups and adequate follow-up can provide strong evidence for causality and are recommended [68]. Fitzhugh et. al. [68] report the results of a natural experiment concerning trails that incorporated many of the recommended standards for assessing causal inference. Studies like this can provide strong guidance for planners and policymakers (see Table A3). This study examined the effectiveness of a newly retrofitted urban trail that was implemented in one neighborhood among many similar neighborhoods with poor connectivity in the community [68]. This trail was designed for a neighborhood lacking connectivity between residential pedestrian infrastructure and nonresidential destinations to increase physical activity among children, adolescents, and adults. The effect of the change in infrastructure was evaluated by comparing the behavior of study participants in the neighborhood that underwent the trail infrastructure improvement with two control neighborhoods that did not undergo any improvement. Some of the recommended design standards used include the following: all neighborhoods had similar characteristics at baseline; intervention and control neighborhoods were well matched; data on all communities were assessed before the intervention; and physical activity was assessed with 2-hour direct observation of trail use. This trail infrastructure improvement significantly increased physical activity in the intervention neighborhood, while physical activity declined significantly in control neighborhoods. The pre-and post-intervention improvements for physical activity in the experimental neighborhood compared with the control neighborhoods were statistically significant for total physical activity, walking, and cycling [68]. Other trail and built environment improvements identified in the literature may be considered when developing future interventions to increase physical activity among youth. For example, reducing road traffic is associated with increased physical activity in neighborhoods [87], and park improvements such as adding a new path/trail, have also led to increases in physical activity [49].

Another example of the type of research that could advance knowledge related to trail use by youth from under-resourced communities are studies that examine trail use in conjunction with Safe Routes to Schools programs and policies. More intervention studies have addressed active transportation to school than leisure or recreational walking by children and youth (see Table A2 for a select sample of studies) [50,51]. However, further research and interventions are needed to address trail use and active transportation together with youth-oriented programs, such as Boys and Girls Clubs, YMCA’s/YWCA’s, Parks and Recreation program offerings, Community Youth Centers, and after school programs. The body of research pertaining to active travel to schools can serve as a model for the type of research that is needed to advance knowledge about how to effectively connect trails with programs for youth that, in turn, promote and facilitate increased use of the trails themselves.

An example, of how a “program” related to active travel to school was incorporated into a comprehensive community intervention illustrates the type of research that can lead to effective trail use programs in under-resourced communities. This study, in Jackson Michigan, was supported by a Robert Wood Johnson Foundation’s Active Living by Design grant. Jackson, a community of 35,000 residents (with 15.2% of families below the poverty level) developed a community intervention using the Active Living by Design’s 5P Model (Preparation, Promotion, Programs, Physical Projects, Policy). The intervention included partnership building and a multilevel community intervention that focused, in part, on creating safe transportation routes to encourage walking and biking to school and to worksites. Baseline measures were obtained in 2003 prior to intervention implementation and again post-intervention in 2005/2006. Findings indicate that there was a change in attitudes toward active transportation (i.e., 8% increase in children who thought walking to school was “safer” post-intervention), intentions to try active transportation (i.e., 43% of Smart Commute Day participants “would” smart commute more often post-event), and, importantly, increased physical activity (i.e., the percentage of students walking to school more than doubled at three of four intervention schools). In addition, a community level observational study was conducted at 10 locations in the city in 2005 and 2006. The number of people seen using active transportation increased from 1028 in 2005 to 1853 people in 2006 (a 63% increase).

In August 2018, the Community for Preventive Services Task Force recommended interventions to increase Active Travel to School, such as the Safe Routes to School (SRTS) program, based on sufficient evidence of effectiveness that the interventions increase walking among students and reduce risks for traffic-related injury [83]. The Task Force also found that the economic benefits exceed the cost for active travel to school interventions. Active travel to school interventions were made possible, in part, due to funding opportunities that may also be needed to support other programmatic activities to increase routes or trails use among youth. In the U.S., the Safe Routes to School program launched in 2005 resulted in more active transportation to school with fewer injuries [90]. Safe routes to school projects are currently eligible under the Transportation Alternatives Set-Aside and the Surface Transportation Block Grant Program for funding. SRTS funds may be used for infrastructure-related projects, which may include sidewalk improvements, off-street bicycle and pedestrian facilities (for example, trails), traffic-calming measures, bicycle lanes, and bike racks [83,84], and for non-infrastructure related projects, which may include student and parent education, public awareness campaigns, and traffic enforcement [82]. The Federal Recreational Trail Program provides funds to the States to develop and maintain recreational trails and trail-related facilities that can support additional trail infrastructure in under-resourced areas [83]. The Federal Recreational Trail Program may be one source of support for bridging trails with programs promoting their use by youth in under-resourced communities [82]. Such infrastructure-related projects could also be useful targets for further research addressing how to increase trail use and physical activity in under-resourced populations [91].

## 4. Discussion

The primary purpose of this review was to identify effective programs for increasing trail use among youth from under-resourced communities, in order to address disparities related to the underrepresentation of trail users from different racial and ethnic groups and/or low-income neighborhoods and communities more broadly. Unfortunately, this review did not find studies evaluating programs that promoted the use of trails among youth from under-resourced areas. In fact, the review highlights a lack in our knowledge on how to promote and increase the use of trails by youth regardless of their socioeconomic status or where they reside. Connecting safe (pedestrian, bicycle, or public) transportation systems to destinations or places where youth reside in programs where they may congregate to use trails to pursue outdoor recreational opportunities and enjoy nature has not been systematically evaluated. It is possible that such programs exist and have just not been studied using experimental designs and methods. This highlights a critical research gap and lack of knowledge about how to intervene to connect youth, and/or programs for youth, to trails to increase their physical activity as a health-enhancing behavior.

To address this research gap, it is recommended that natural experimental research designs be considered to provide insights into possible causal factors that may link youth programs to increased use of trails. Recent workshops and reports of evidence-based reviews, focused on enhancing methods for the evaluation of natural experiments, have provided a clear set of recommendations (e.g., intervention and control neighborhoods well-matched, baseline characteristics similar at intervention outset) on how to improve current research designs and the data resources needed for such research. Furthermore, a September 2018 Federal Partners meeting organized by the National Institutes of Health, Office of Disease Prevention addressed approaches to enhance collaborative efforts in the area of the evaluation of the effects of obesity policy and programs across federal health, transportation, housing, environmental and park agencies—highlighting the need for cross-sectoral work to achieve progress in this arena [92]. These workshops, reports, and meetings hold promise for advancing our knowledge related to trails use among different segments of the populations and addressing disparities among trail users.

Active travel to schools, such as Safe Routes to Schools, has been found to be effective in increasing active commuting to schools among youth. The body of research that represents the evidence for the effectiveness of active commuting to schools or Safe Routes to Schools is also highlighted in this review as an example of what may advance science and knowledge about attracting more youth participants, including those from under-resourced neighborhoods and communities, to use trails for recreational physical activity.

Since trail features themselves may influence trail accessibility and use among youth, a secondary purpose of this study was to identify trail features that may be considered during efforts to connect safe trails to programs that promote their use. Studies have found that it would be beneficial for such programs to be in close proximity to trails [93,94,95,96].

It may also be necessary to provide safe active commuting (walking, bicycling or public) transportation system options to connect with trails that are not in close proximity to youth programs. Trails are one form of the built environment that may influence recreational and transportation physical activity among youth and adults, and this review did identify a variety of built environment features that may impact trail use, such as access to and proximity of trails.

Features of destinations that are attractive among youth may also inform the planning of programs to promote trail use among this population. For example, parks, greenspaces, and open spaces may be enhanced by programs to accommodate youth recreational trail users and their connectedness to opportunities to recreate and enjoy nature. Providing lighting, improving perceptions of, and actual safety [38], and increased accessibility are examples of features important to both trails use and destinations where youth programs are located.

Other secondary purposes of the review were to identify the benefits of, and barriers to trail use that may inform the development of programs for our population of interest. Insights into the barriers associated with using trails, or sidewalks, include neighborhoods with high crimes, automobile traffic congestion making walking unsafe [75], lack of exposure and opportunities to access trails, costs, lack of transportation, lack of role models using trails, and institutional discrimination [51]. It will be beneficial to plan in advance how to address barriers that deter youth from under-resourced areas from using trails for physical activity. These efforts will most likely benefit greatly by programs being centered in destinations where people from different races/ethnicities, backgrounds and cultures intermingle and socialize, since people who do not see role models that look like them attending physical activity programs or pursuing recreational opportunities will be unlikely to initiate involvement and/or sustain participation in recreational physical activities.

Programs designed to support trail use among youth, including youth from under-resourced communities, may also benefit by capitalizing on promoting the social, mental, and physical health benefits that can be derived from trails use, outdoor recreational pursuits, and enjoying nature. Doing so may optimize program participation and success. One study was located that evaluated health benefits in our review reporting a psychological outcome, indicating that youth having greater access to greenspaces also had higher self-esteem and health-related quality of life. There is a need to evaluate the benefits of trail use among youth that may be associated with increases in physical activity as a part of outdoor recreation or connectedness with nature.

## 5. Conclusions

The primary aim of the paper was to identify evidence-based trail studies that promote and/or increase trail use among youth from under-resourced communities. Few intervention studies using trails to increase physical activity among under-resourced youth were identified in this review. Clearly, more studies need to be conducted using access to trails as interventions to promote trail-use among youth. In most of the adult trail studies using trails as the intervention tool provide limited insight to this specific segment of the population. How to increase the use of trails among youth for purposes other than active commuting to school, such as using trails to enjoy nature and outdoor recreational pursuits, is unknown and needs further attention. Planning, implementing and evaluating the use of trails to increase physical activity among youth, and programs specifically designed to facilitate the use of trails for outdoor recreation, could benefit from greater attention from researchers and practitioners in the future. Efforts to directly address disparities related to trails use among youth from low income, often racially/ethnically diverse, under-resourced neighborhoods and communities are especially needed.

The recent reports from the Community Preventive Services Task Force [4] about the influence of the built environment on physical activity and on active travel to schools provide evidence of just how rapidly knowledge about physical activity and the built environment is evolving. These reports highlight the increased rigor of research designs now in use to evaluate the effects of the built environment on physical activity behaviors. However, systematic reviews related to physical activity and the environment do not provide knowledge about “how to” implement effective programs that may increase the use of the built environment especially among select population groups of interest, such as youth from low income, diverse, and/or under-resourced communities.

The Task Force physical activity-built environment recommendation noted the need for ensuring that a transportation system (e.g., pedestrian trails, bicycle routes, or public transit) connect to the built environment and land use destinations, such as a facility housing a program (e.g., Boys Club and Girls Club of America, YMCA/YWCA, or school club). However, this review of the scientific literature did not identify an effective trail use program for youth from under-resourced communities, that is housed in a destination or setting such as a school, YMCA/YWCA, or Boys and Girls Club.

## 6. Practical Applications

To make advances in this area, future research and practice efforts are needed to establish programs designed to help more youth enjoy nature and outdoor recreational opportunities. Future research efforts should focus on developing interventions to promote trail use rather than cross-sectional studies limiting causal inferences. This research and practical work should incorporate an evaluation of the intervention and programs’ impact on increasing trail use and assessing other outcomes of interest to expand the knowledge base in this under-studied area that can also then be replicated. 

Practice-based programs may also provide data on feasibility, even if they have not been evaluated using a well-conducted experimental study design, or published in a peer-reviewed journal. Practice-based programs accompanied by evaluation data may also inform and influence better designed future experimental research. A companion brief based on a review of programs and practices related to trails use among youth from under-resourced communities or neighborhoods, is being developed and when completed will be available on the NCCOR website (https://www.nccor.org). 

## Appendix A

**Table A1:** Correlates of Physical Activity and Features of Transportation Systems (Routes) and/or Built Environment and Land-Use Destinations or Both Routes and Destinations that May Support Planning and Implementation of Programs to Increase Trail Use among Youth from Under-Resourced Communities.

**Section A. Correlates of physical activity/trail use and features of transportation systems (routes) (e.g., access to trails, walking paths, bicycle paths, sidewalks) that may inform trail use programmatic efforts to increase physical activity among youth.**				
**Routes**	**Citation**	**Year(s) of Data**	**Data Collection Methods/Measures**	**Results**
Pathways	[26]	1999–2011	Review	Pathways for bicycles and walking were more likely to promote regular physical activity in children.
				Physical activity
Trail	[30]	1989–2004	Review	Having “highly walkable” neighborhoods were more likely to encourage people to walk for transportation. For people who reported using the trail in this study, over half of them increased their average amount of walking.
Trails	[31]	2001	RDD Telephone Survey (Perceptions of Environmental Support Questionnaire); GIS; BRFSS Physical Activity module	Participating in more physical activity/walking was found to be associated with lower BMI levels. The availability of trails was associated with twice the odds of being overweight as opposed to obese for users that do not meet the national physical activity recommendations.
				BMI Trail use; walking for active commuting
Development of a New Trail	[37]	2008–2011	Intercept survey	A majority of the participants traveled to the trail using active transportation (69.7%), and the typical user was found to less likely to be a college graduate. Overall, 89.7% of participants reported using the trail for recreational activities.
				Active travel to trail for recreational activities
Trail use	[39]	2006–2009	Intercept survey	Younger adults, men, White, and those with some graduate school education were more likely to perform physical activity on the trail. Higher PA was also associated with usage in cooler months, travelling to the trail in a motorized vehicle, and going with others.
				Trail users; Physical activity
Trail-proximity	[41]	1980–2008	Review	The further a trail was from a participant’s home, the less frequently it was used. Education and income were both positively associated with trail use.
				Trail use
Paved streets Bicycling lanes [Proper lighting]	[93]	1993–2012	GENEActiv accelerometers. IPAQ survey. Physical Activity Resource Assessment (PARA).	When observing which factors influenced the physical activity levels of Brazilian children, it was found that paved streets, bicycling lanes, and proper street lighting were important features that increased the frequency of activity.
				Physical activity
Roads Street conditions	[94]	2007–2013	Review	Roads and street conditions are important indicators of active transportation and physical activity in youth.
				Physical activity; Active transportation
**Section B. Correlates of physical activity/trail use and features of the built environment and land use destinations (e.g., parks, park enhancements, greenspaces, open spaces) that may inform trail use programmatic efforts to increase physical activity among youth.**				
**Destinations**	**Citation**	**Year(s) of data**	**Data Collection Methods/Measures**	**Results**
Parks Playgrounds Playing fields	[28]	2004–2008	SOPARC	45 parks in a Southeastern community were evaluated to identify the activity settings used by boys and girls. There were fewer girls observed in the parks than boys, but the most commonly used structure by both genders was playgrounds. Playing fields were popular among both, but more frequently visited by boys. This setting was associated with greater vigorous physical activity. Observations of seven of eight activity settings in the 45 parks indicated a greater frequency and percentage of white youth observed in comparison to minorities.
				MVPA
Neighborhood Features Parks Recreational facilities Retail, ratio of floor space	[34]	2006	GIS; Actigraph accelerometer	Girls were more likely to participate in physical activity when there were a higher number of recreational facilities and parks in the surrounding environment. Boys’ physical activity was shown to increase with the ratio of retail floor area.
				MVPA
Greenspace	[56]	2014	Kid-KINDL questionnaire	More access to greenspace and having fewer siblings were associated with better health and quality of life among children. This availability of greenspace was also linked to greater self-esteem.
				Health and Quality of Life; Self-esteem
Destinations: Diverse housing types Mixed land use Housing density Compact development patterns Open space	[95]	2000–2009	Review	Diverse housing types, mixed land use, housing density, compact development patterns, and levels of open space were the five factors that affected the amount of physical activity seen in an environment. Factors such as the proper planning of built environment features are essential to promoting physical activity.
				Physical activity
Park playground-proximity	[84]	2006	GIS; Environmental Assessment for Public Recreation Spaces (EAPRS)	Having a park playground within 1 km of the home significantly decreased a child’s odds of being at risk or overweight. These children were five times more likely to be a healthy weight than children who did not have a playground in the nearby park.
				Healthy weight / Overweight
Greenways	[74]	2017	Adapted Safe Routes to School survey	Greenways make it easier for students to participate in active transportation via walking or cycling due to the improved infrastructure conditions.
				Active transportation
Home School Green spaces, including parks, wooded areas, and vacant land	[96]	2012	Accelerometers GPS data loggers Questionnaires	This study evaluated the MVPA of rural, urban, and suburban students. Rural students were more likely to get most of their MVPA at school, but were three times less likely to partake in this activity than urban students. For both urban and suburban students, active commuting made up a large percentage of their average MVPA. MVPA among youth occurs at locations other than home, school, or through active transportation. Green spaces, including parks, wooded areas, and vacant land are also locations where youth attained
				MVPA.
Parks	[97]	1994–1995	Data from Wave I of National Longitudinal Study of Adolescent Health; GIS	The availability of parks was associated with benefits such as increased participation in active sports. Females were more likely to participate in wheel-based activities, such as cycling.
				PA: Sports participation; Cycling
Parks Park enhancements—Fitness zones	[98]	2000	SOPARC	Fitness Zones were added to some parks and not added in others (control). Usage of the park increased with the addition of the new equipment, and there are higher estimates of energy expenditure.
				Park usage; energy expenditure
Park enhancements—new equipment added Amid decrease in programming activities	[99]	2003, 2004, 2006, 2008	SOPARC	This study evaluated the effect of adding park improvements. Contrary to expectations, it was found that physical activity decreased in both parks with new equipment and those without. This decline was attributed to a decrease in programming during that period.
				Physical activity
**Section C. Correlates of physical activity/trail use and features of transportation systems and built environment and land use destinations that may inform trail use programmatic efforts to increase physical activity among youth.**				
**Routes and Destinations**	**Citation**	**Year(s) of data**	**Data Collection Methods/Measures**	**Results** **Physical Activity Outcome(s)**
Routes: Intersection density Public transport density Destinations: Residential density Number of parks	[43]	2002–2011	International Physical Activity and Environment Network (IPEN); Actigraph accelerometer; GIS	Residential density, intersection density, public transport density, number of parks significantly associated and linearly related to physical activity. On average, participants had about 37 min/day of MVPA.
				MVPA
Routes: Higher traffic Incomplete sidewalks Intersection density Destinations Residential density Recreational open spaces—number of and proximity	[44]	2008–2012	GIS	Children who lived closer to recreational open space had additional opportunities for physical activity and thus also had lower BMI z-scores. On the other hand, fewer nearby recreational open spaces, high traffic, sidewalk completeness, intersection density, and less residential density were associated with higher BMI z-scores.
				PA and BMI z-scores
Routes: Neighborhood bicycle or walking trails Ease of walking or biking to transit Destinations: Neighborhood safety Neighborhood aesthetics (more trees, interesting things to look at, and lack of garbage or litter) Number of activity facilities Destinations of interest within walking distance of homes Parental permission to walk in the neighborhood, take public transportation, or walk/bike to transit	[21]		Youth Survey	Examined the test-retest reliability of a survey designed for youth to assess their perceptions of physical environmental factors and transportation. Evaluated the associations of these perceptions with both physical activity and active transport to school Neighborhood walking trails; destinations within walking distance from home, safety, aesthetics, facilities, and being able to walk or bike for transportation were related to physical activity, and in some cases to Active Transport (ATS)
				Physical activity; Active Transport to School (ATS)
Route: Multi-use paths Destinations: Parks	[61]	2011–2013	Actical Z accelerometers; STEAM study (Spatial–Temporal Environment and Activity Monitoring)	Neighborhoods with parks that contain sports fields and multi-use paths were found to significantly increase the average daily MVPA of children outside of school. This can be partially attributed to the fact that paths allow for active transportation.
				MVPA
Routes: Walking paths Destinations: Public toilets Lighting Park Proximity	[100]	2006	GIS	Having features such as public toilets, lighting, more than 25 trees, and walking paths were all linked with higher park usage. For those that used the park, 27% of participants used the park closest to home for physical activity.
				PA-park usage
Routes: Trails Destinations: Multi-use zones Lakefront zones	[101]	2009–2010	Modified Alfonzo’s Hierarchy of Walking Needs; St. Louis University Environmental Checklist Audit Tool.	The study found that 64.4% of youth participants were moderately active, while only 2.9% were vigorously active. The groups that participated in activity the most were males, younger children, and African American children. The type of environments used most frequently were trails and multi-use zones (80.5%) and lakefront zones (45.1%).
				MVPA
Routes Traffic Destinations Beach close to a school Nearby park	[88]	2005	Physical activity location mapping used with open-ended questions	Children reported enjoying to partake in physical activity the most at a beach close to school and a nearby park. The reasons for this preference included being able to participate in sports in that location and being able to have fun there. Common barriers to physical activity included schoolwork, weather conditions, traffic, and strangers. Only 23% of students walked for transport, and less than 2% biked to their favorite places. Overall, 39% of males and 46% of females used a car for transport. Physical activity; Sports participation
				Physical activity
Routes: High traffic Destinations” Number of trees Lighting	[85]	2005–2008	MEGAPHONE special data infrastructure; Questionnaires	Neighborhood features that increased children’s’ feelings of neighborhood safety were a high number of trees and adequate lighting. The feature that decreased this feeling of safety was a high proportion of minorities in the surrounding area. Parents showed similar trends but were more likely to associate high traffic and a lack of community involvement with decreased safety.
				Perception of safety
Routes: Infrastructure for active transportation and recreation Safe routes to all destinations Destinations: Parks Sports centers Neighborhoods with mixed planning	[102]	1989–2009	Review	The availability of infrastructure that allows for active transportation and recreation promotes greater physical activity. Additionally, parks and sports center were associated with higher MVPA in youth. Neighborhoods with mixed planning were more likely to have residents that participated in greater out-of-school physical activity. Although the focus to date has been on providing safe routes to schools, greater attention could be given to creating safe routes to all local destinations such as shops and shopping centers, which would enhance the quality and walkability of local environments for all residents, including children, adolescents and older adults.
				MVPA
Poor quality of sidewalks Playgrounds Soccer fields Pools	[103]	2012	2010 Census; SOPLAY; Physical Activity Resource Assessment (PARA)	Neighborhoods with poor walking conditions and low-income residents were associated with less vigorous activity. Youth were most likely to use playgrounds, soccer fields, and pools. Low-income neighborhoods were more likely to have lower-quality sidewalks, and this was related to fewer odds of physical activity.
				Physical activity
Routes: Sidewalks Destinations: Playgrounds Picnic areas	[89]	2007	GIS; SOPARC	Factors that encouraged physical activities included playgrounds, sidewalks, and picnic areas. Major inhibitors to park use included crime and racial heterogeneity of the neighborhood.
				Park use
	[98]	2000	SOPARC	Fitness Zones were added to some parks and not added to others (control). The usage of the park increased with the addition of the new equipment, and there are higher estimates of energy expenditure.
				Park use
	[43]	2002–2011	International Physical Activity and Environment Network (IPEN); Actigraph accelerometer; GIS	Residential density, intersection density, public transport density, number of parks significantly associated and linearly related to physical activity. On average, participants had about 37 min/day of MVPA.
				MVPA

**Table A2:** Select Studies Evaluating Active Travel to School or Safe Routes to School: An Evidence-based Intervention That May Serve as a Model for Connecting Trails to Youth Trail Use Recreational Programs.

Active Travel to School or SRTS	Citation	Year(s) of Data	Data Collection Methods/Measures	Results Physical Activity Outcome(s)
	[83]	2018	Review	Sufficient evidence of effectiveness supporting for Active Travel to School (ATS) is available that supports increased walking among students and reduce risks for traffic-related injuries.
				Walking; traffic-related injuries
	[81]	1969–2001	National Personal Transportation Survey	There has been a decrease in active transportation to school among children, resulting in lower levels of physical activity. It was encouraged that schools should be placed within walkable neighborhoods.
				Physical activity related to active transportation
Active travel to school or SRTS and Participation in physical activity before school Daily moderate-intensity physical activity	[104]	2004	BMI measurements; Self-Administered Physical Activity Checklist (SAPAC); Parent Questionnaire	Students who walked to school had a lower BMI in the post-test analysis. This association was also seen for children who participated in physical activity before school and daily moderate-intensity activities.
				BMI; findings may differ by sex
	[105]	2000–2004	GIS; 2000 U.S. Census; Database of elementary, middle, and high schools in the U.S. coded by geographic location	This study provided evidence that showed that over 65 million urban residents could benefit from a SRTS project to improve walking and cycling conditions. As a result, adults and children would be more likely to participate in physical activity in these areas.
				Physical activity
	[106]	2007–2008	PLAY-On questionnaire	Participating in moderate to intense physical activities decreased a student’s odds of being overweight. This activity could come from opportunities for active transportation to school, and/or that provide student access to a variety of recreation facilities during school hours. The main indicator of a student’s odds of being overweight was their grade level.
				MVPA
	[107]	2010	Interviews; 2006 Census	Active transportation to school was more common among children in suburban or semi-suburban neighborhoods as opposed to those in urban locations. The presence of neighborhood amenities increased this level of activity.
				Active transportation to school
	[108]	2001–2010	Motor vehicle crash data; ArcGIS	The implementation of the SRTS program in New York City significantly decreased the rate of pedestrian injury among school-aged children.
				Injury rate

**Table A3:** Longitudinal or Quasi-Experimental Studies Related to Physical Activity or Trail Use.

Routes and/or Destination Intervention Features	Citation	Year(s) of Data	Data Collection Methods/Measures	Results Physical Activity Outcome(s)
Longitudinal study design Route: Bicycle and walking trails in neighborhoods Volume of traffic Destinations Neighborhood Lighting—well-lit streets Neighborhood outdoor play Ease of access to 14 facilities or destinations including Parks Playing fields Swimming pools Recreation center or YMCA/YWCA.	[67]	2002–2011	Actigraph accelerometer, Questionnaire	Non-school physical activity declined for both boys and girls from the 6th to the 8th grade. The facilities that were easiest to get to were parks, playing fields, paths or trails, and swimming pools. A majority of the sample said that there was crime in their neighborhood, as well as, enough traffic to make it hard to walk. Study—youth participants (53.5% white; 18% Black; 19.1%; Hispanic; 4.8% Asian, Native Hawaiian or Pacific Islander) reporting that children do not play outdoors in the neighborhood, that their neighborhood was well lit, and that there were trails in their neighborhood at baseline, were each associated with significant decreases in non-school Met-Weighted -MVPA at 2-year follow-up. No neighborhood measures were associated with sedentary behavior. MET-weighted MVPA (MW-MVPA), and sedentary behavior.
Quasi-experimental design, a prospective natural experiment. Routes: An urban greenway/trail	[68]	2005, 2007	Pedestrian count; Survey; Direct observation of Physical Activity	An urban greenway/trail was retrofitted in a Knoxville, TN neighborhood that lacked connectivity. The experimental neighborhood (17.7% minority ethnicity); was compared to two matched-control neighborhoods. Two-hour counts of physical activity increased from 2005 to 2007 in the intervention neighborhood but decreased in the control neighborhoods. Total physical activity; walking and cycling—active travel to school
Safe transportation routes to schools and work sites.	[69]	2003, 2005, 2006	The study methods used the Active Living by Design’s 5P Model (Preparation, Promotion, Programs, Physical Projects, Policy), to guide intervention planning and implementation.	This study highlights the results of a Robert Wood Johnson Foundation’s Active Living by Design grant that supported partnership building and a multilevel community intervention in 2003 in Jackson, MI (Jackson has more families below the poverty level (15.2%) than the average in Michigan and the United States; race/ethnicity demographics not reported). The safe physical activity project, which focused, in part, on creating safe transportation routes to encourage walking and biking to school and to worksites, was shown at follow-up (2005/2006) to change attitudes toward active transportation (8% increase in children who thought walking to school was “safer” post-intervention), intentions to try active transportation (43% of Smart Commute Day participants “would” smart commute more often post-event), and increased physical activity (the percentage of students walking to school more than doubled at three of four intervention schools). In addition, a community level observational study was conducted at 10 locations in the city in 2005 and 2006. The number of people seen using active transportation increased from 1028 in 2005 to 1853 people in 2006 (a 63% increase). Walk to school; Active transportation to work
Pre-test post-test quasi experimental study. Evaluating a marketing/media campaign to promote trail use. Routes: Trails	[70]	2011–2012	Infrared monitors; Manual audits	The purpose of this study was to evaluate whether marketing of specific trails can increase their use. A Las Vegas, NE, a media campaign to promote a new searchable trails website, as well as the use of the southern Nevada trail system, was conducted. The media/marketing campaign of trails included radio, print, online ads, billboards, and signs on top of gas pumps. The media campaign ran for 8 weeks and was shown to increase trail use on 8 of 10 trails studied from an average of 3.91 users per hour to 5.95 users per hour. For the two trails whose use declined, it is possible that with new information available regular users of those trails switched to other, higher quality trails. The ads targeted women ages 18-54 and parents of children age 8–15, although it is unclear how the researchers targeted these specific audiences. Trails Use
Pre-test Post-test quasi-experimental design Routes: Addition of a new walking path—creating a safe transportation route Destinations: Playground, in a low income African-American neighborhood.	[71]	2006, 2008	Door-to-door surveys; Self-report Direct Observation; SOPLAY	A 6-block walking path and a school playground were installed in a low-income, largely African-American intervention neighborhood, in New Orleans, LA. Physical activity levels were measured in the intervention neighborhood and in two matched comparison neighborhoods by self-report, using door-to-door surveys, and by direct observations of neighborhood residents outside before and after the interventions. Self-reported physical activity increased over time in most neighborhoods. The proportion of residents observed who were active increased significantly in the section of the intervention neighborhood with the path compared with comparison neighborhoods. There was no significant pre-playground intervention post-intervention findings noted for physical activity measures. Path use—walking and biking; Physical activity
A natural experiment, pre-test post-test quasi-experimental non-control design Conversion of a rail trail to a multi-use trail	[65]	2000	Telephone survey of adults ≥18 years. Survey questions asked about: Leisure activity Walking and bicycling Moderate and Vigorous Physical Activity Transportation Activity Trail Use	A railway of >23 miles was under development for conversion to a multi-use trail. A segment of the trail was evaluated by randomly selecting and telephone interviewing adults living within 2 miles of the planned trail before trail construction began and approximately 2 months after completion of construction. For the total population, living nearby the part of the trail segment evaluated, 41.2% of the residents were black and 47.3% were white. At follow-up, 11.0% of respondents had not heard of the trail, and 23.1% had heard of the trail and had used it at least once. Leisure activity, leisure activity near home, moderate activity, vigorous activity, and walking for transportation did not significantly change for those who used the trail compared to those not using the trail. This prospective study of the building of a multi-use trail did not demonstrate an increase in physical activity among adults. Trail use and other physical activity
Routes: Rail Trail	[32]	2000–2001	Telephone surveys of adults of ages 18–55 years	The Sydney, AU Road and Traffic Authority (RTA) completed the construction of a 16.5-km-long Rail Trail cycleway in Western Sydney in December 2000. A short-term local promotional campaign to increase awareness and use of the Rail Trail was conducted. The media campaign included local press advertisements about the newly constructed Rail Trail, and maps of the trail in English and six other languages. There was a significant increase in unprompted Trail awareness, but post-campaign awareness was low (34%). Pre and post-campaign telephone surveys were conducted with adults 18–55 years of age living in an “inner” area, within 1.5 km of the Trail, and an “outer” area, bike-owners only, 1.5–5 km from the Trail. Trail usage was higher among bike-owners than pedestrians and was moderated by proximity to the Trail. The campaign reached and influenced cyclists in the inner area. Inner cyclists increased mean cycling time by 0.19 h (SD _ 1.5) while outer cyclists decreased cycling, time (_0.24 h, SD _ 1.6).
Routes Way-finding, and incremental distance signage along trails	[66]	2011–2012	Infrared monitors placed on the trails for three periods of 7 days, during a 1-year timeframe.	Comparisons were made between pre-, mid-, and post-intervention usage rates on six trails where signage was added, to usage rates on four control trails. The signage was added to trails after a previous marketing campaign was used to effectively promote and increase trail usage on 8 of 10 trails studied. Wayfinding and incremental distance signage were associated with about a 33% increase in trail use (mean users per hour increased 31% for control trials and 35% for the trails with signage), but the total increase did not vary between the intervention and control groups. The marketing campaign intervention that occurred prior to the wayfinding and distance signage intervention that followed, may have influenced findings.
